# Vaginismus in Assisted Reproductive Technology centers: an invisible population in need of care

**DOI:** 10.5935/1518-0557.20180013

**Published:** 2018

**Authors:** Maria do Carmo B de Souza, Marcia C G Gusmão, Roberto A Antunes, Marcelo M de Souza, Ana L S Rito, Paloma Lira, Ana C A Mancebo, Maria A Tamm, Tatiana R Panaino, Maria J Bahia

**Affiliations:** 1 Fertipraxis Reprodução Humana, Rio de Janeiro, Brazil

**Keywords:** Vaginismus, dyspareunia, ultrasonography, *in vitro* fertilization

## Abstract

**Objective:**

Genital and sexual pain is still neglected. Consequences may be dramatic, since infertility and sexual dysfunction may be reciprocally linked. This is the first study to focus on the identification of cases of vaginismus in the ART scenario and on the introduction of intra-cycle interventions as part of a comprehensive, integrated and patient-centered perspective.

**Methods:**

This observational prospective study looked into 425 IVF/ICSI cycles and 226 frozen embryo transfers carried out from January 1, 2015 to December 31, 2016, and found seven cases of vaginismus. Within a six-month period, a questionnaire placed on SurveyMonkey was sent twice to 228 ART centers in Latin America. The purpose was to learn how often cases of vaginismus were found in ART centers and the perceptions around the presence of this condition.

**Results:**

The few centers that took the time to answer the questionnaire (24/10.5%) stated that the number of cases in which they had trouble performing control ultrasound examination or needed to perform transfers with patients under sedation was not significant. Although 81% agreed that the incidence of these conditions is low, no references were made to cases of vaginismus, dyspareunia or sexual dysfunction. Our multidisciplinary team found seven cases of vaginismus, involving women with higher education degrees with a mean age of 37.8 years and married for a mean of four years. Although two reported they were able to tolerate intercourse, all reported undergoing treatments such as using vaginal dilators (3), psychotherapy (4) and psychiatric care (1). The care provided by the staff was designed to mitigate patient suffering.

**Conclusion:**

Gentle care and sensitive listening should be integral components in the work of multidisciplinary teams to identify women with vaginismus and offer couples better quality treatment.

## INTRODUCTION

"In women, what we call the womb or uterus is an animal inside them that has the appetite to bear children; And, when it is long without fruit, this animal is impatient and supports this state with difficulty; Errs through the whole body, obstructs the passage of the breath, prevents the breath, throws the extreme anguish and causes other diseases of all kinds.

Plato, Timaeus, (cited in Nasio, 1991)

Pain is a complex perceptive experience. Bearing biologic, psychological and relational significance, it gains importance as it becomes chronic. Lifelong and acquired genital and sexual pain is still neglected in a consistent percentage of women (Graziottin *et al.*, 2015). The consequences may be dramatic, since infertility and sexual dysfunction may be reciprocally linked. Causes of sexual dysfunction leading to infertility include erectile dysfunction, Peyronie's disease (abnormal penile curvature), low libido and ejaculatory disorders in men, and genito-pelvic pain/penetration disorder (GPPPD) and low sexual desire in women (Berger *et al.*, 2016).

Reviews usually address GPPPD as a broad disorder that includes vulvodynia and vaginismus, treated with topical lubricants and moisturizers (Berger *et al*., 2016). There is also a Cochrane Review about various therapeutic strategies for vaginismus, such as sex therapy and desensitization (Melnik *et al*., 2012).

Vaginismus, with its associated defensive contraction of paravaginal muscles when intercourse is attempted, is credited to be the pelvic expression of a more general muscle defense posture, associated with a variable phobic attitude towards coital intimacy. This can result in intercourse avoidance in more severe cases, while milder manifestations may cause dyspareunia. Psychosexual factors such as loss of libido and arousal disorders may be present in sexual pain related disorders. These may contribute to the worsening of coital pain over time, in isolation or association with couple infertility. And, of course, vaginismus "per se" may be the etiology of infertility. Protti & Rodrigues Jr (2008) categorized vaginismus as primary (penetration by the penis and other devices is not possible); secondary (the dysfunction appears when penetration is perceived as a threat); selective (only with some partners); and non-selective (occurring with any partner).

The clinical approach to GPPPD aims at diagnosing biological, psychosexual and context-dependent etiologies. Patients with GPPPD attempting pregnancy might be present in ART centers. There is very little data in the literature on women with GPPPD and their performance on ART cycles. An ART program implicates vaginal manipulation during transvaginal ultrasound (TVUS) examination in ovarian stimulation, oocyte retrieval, and embryo transfer, all of which might pose a significant challenge to these patients. This is the first study designed to identify cases of vaginismus in an ART clinic and to consider intra-cycle interventions in an ART center as part of a comprehensive, integrated, patient-centered perspective.

## MATERIAL AND METHODS

This observational prospective study enrolled the patients involved in 425 IVF/ICSI cycles and 226 frozen embryo transfers performed from January 1, 2015 to December 31, 2016. The patients were interviewed by their personal physicians and the chief nurse (to receive medication-related advice), and talked to three other physicians during control ultrasound examination. The ultrasound operator started by reading the patients' records before examining them, then introduced himself to the patients, informed them that they had a transvaginal ultrasound scheduled, and asked them if they had any concerns. A psychoanalyst assigned to the team worked as a patient liaison and was present before and after oocyte retrieval procedures and embryo transfers. Seven cases of vaginismus were found in the clinic during the study period, through calm listening and open-minded interviews with the staff (Rosenthal, 2014). Detected cases were flagged for extra-individualized care during ART and the information was passed on to the anesthesiologist.

In July of 2016 a questionnaire entitled "A glance at women undergoing assisted reproductive technology procedures" was posted on SurveyMonkey^®^. The questionnaire was first tested with the staff and physicians working at the clinic. Then it was sent to 228 ART centers in Latin America (131 REDLARA centers plus 97 centers cited in the official website of the Brazilian Society for Assisted Reproduction, SBRA). The goal was to learn how often patients with these conditions were seen at ART centers and the possible perceptions around their presence. The questionnaire featured 10 direct questions written in Portuguese and Spanish, and was presented and signed by the main author (Annex).

The first question was designed to estimate the size of the responding center. The centers were asked to estimate the proportion of patients that had trouble during transvaginal ultrasound examination and the perceived causes, in addition to whether the gender of the ultrasound operator was an issue. Other questions probed into the proportion of embryo transfers performed under sedation and the reason for sedating the patients. Respondents were also asked to share their impressions about the questionnaire and the topics it addressed.

Six months later, in January of 2017, the questionnaire was resent to the centers that did not answer it the first time.

## RESULTS

### A - Case reports

1. MTC, 42, married to a man aged 47, with two prior attempts at IVF in another fertility center, described it as "a very difficult experience." She had her first cycle in 2013, with three transfers. The first ended in a miscarriage (Down syndrome) and the second and third did not result in pregnancy. Her husband was a quiet man. He only said that he wanted his name to be correctly spelled. They came to the center in August of 2015 for a new cycle and to undergo PGS. During consultation with the nurse she reported a history of vaginismus, which she had omitted to the physician, and asked for sedation during the transfer procedure. She said she could "tolerate" the transvaginal probe, but that the speculum would be impossible to bear since it would remind her of a course of dilation therapy she had had in the past. She asked the nurse to inform the physicians of her condition because she was too embarrassed to do it herself. While sedated for the oocyte pick-up, she strongly tried to adduce her legs when the speculum touched her vagina. She was due for a transfer under sedation, but the couple had no embryos to transfer (two aneuploid blastocysts) and decided to consider implanting donor oocytes.
2. TBB, 38, married to a man aged 39, came to our center asking for IVF (September 2015) because she "did not tolerate vaginal penetration". She was seen by a sexologist/psychologist, and she could bear nothing beyond her husband touching her perineum. It was decided she would undergo pelvic ultrasound examination and her transfers would take place under sedation. In the beginning of the oocyte pick-up procedure, while sedated with Propofol^®^, she broke the disposable speculum with a vaginal spasm. The pelvic contractions ceased only after her psychoanalyst, who was in the room, whispered in her ear: "you are here by choice, and nobody will invade you against your will." Cycle 1 resulted in biochemical pregnancy. During cycle 2, in May of 2016 (frozen eggs from cycle 1 plus the product of cycle 2), she underwent PGS. Prior to aspiration, by request of her gynecologist, a Pap smear was collected, since she had never allowed the placement of a speculum. In the first sedated frozen embryo transfer (FET) with one blastocyst, she got pregnant but had a miscarriage in the first trimester. She had another transfer under sedation in December of 2016 and had a positive pregnancy test. When she returned for ultrasound examination (7 weeks and 1 day), she allowed the use of a transvaginal probe for the first time. To our surprise, the examination was uneventful. Her husband was present, and the two were very happy.
3. LV, 37, married to a man aged 38, came to the clinic in November of 2014, saying she had had "an IVF cycle in September that did not work" in another ART center. A high dose stimulation protocol was attempted in the first cycle (Gonal F^®^ - rFSH- 300/450IU + Luveris^®^ rLH- 75 IU, Cetrotide^®^ for 5 days, Ovidrel^®^). Only two oocytes were retrieved. In her words, transvaginal ultrasound examinations and the embryo transfer procedure felt "horrible." The cycle resulted only in one cleavage state embryo. She "hated" everything. The couple was offered a procedure with donor oocytes. Further examination in our center revealed an antral follicle count (AFC) of 17 and an AMH of 2.22 ng/mL. During the anamnesis the couple reported "difficulty" with vaginal penetration. She added: "I'm OK with the video examination, but I have to relax and breathe as I've learned to do. And the doctor must take it easy. But the thing is nobody would listen to me in the other center." For reasons linked to religion, the couple preferred not to have their embryos frozen. A new cycle was scheduled in our center using an antagonist (Cetrotide^®^) protocol with Letrozol^®^ (5mg per day) plus Pergoveris^®^ (1 vial per day). They decided that no more than four oocytes would be injected with spermatozoa. From eight oocytes, four metaphase II specimens were frozen and four proceeded to ICSI. The strategy resulted in three cleavage state embryos, two transferred in day 3 after ICSI and one vitrified the same day. She got pregnant and had a term elective cesarean section. In May of 2016 they came in for a frozen embryo transfer (FET) with the previously vitrified embryo plus the resulting embryos from the thawed oocytes. However, the cycle did not result in pregnancy. In July of 2016, a new IVF cycle resulted in the retrieval of 11 oocytes. Again, four M2 oocytes were injected and four were vitrified. She underwent a fresh transfer with two embryos in D3 that resulted in an ongoing gestation (3rd trimester). At every ultrasound check she would be given some time "to concentrate," mostly without the husband present, so that she could endure examination with a vaginal probe. In a noteworthy episode, on her last embryo transfer she asked her husband to remain silent. When he tried to comment on the images of her uterus, she said: "I do not want to see you, I do not want to hear you, I want to relax." As she said it, she had a vaginal spasm.
4. TPC, 41, married to a man aged 37, although off contraceptives for seven years, had been unable to get pregnant. They underwent IUI in 2014 and an IVF cycle in March of 2015 in another ART center, neither of which resulting in pregnancy. The patient came to the clinic in April of 2015. Since they lived abroad, her husband would arrive at a later date. During their infertility investigation, she presented a sperm test with asthenozoospermia, which she considered the cause of their infertility problems. Her AMH level was 0,44 ng/mL. They had two IVF cycles in our center, one in December of 2015 and another in March of 2016. Unfortunately they resulted in three aneuploid blastocysts, and no transfer was performed. Before aspiration on cycle 3, she told the psychoanalyst that she had a really difficult time during penetration. According to her, they could have intercourse but it was always painful. There was an untold past of sexual abuse, depression treatment, and symptoms of suffocation. Her husband had erectile problems.
5. PGB, 34, had been married to a man aged 34 for five years in September of 2016, but had never allowed penetration because of great pain. However, the couple stressed the fact that they loved each other. She said she was unable to undergo gynecological examination. She had previously tried dilation therapy, but could not tolerate it. The prospect of having an anatomical obstruction scared her. During her first visit, it was pointed to her that she had a functioning vagina, since she reported having regular cycles and no dysmenorrhea. A few visits later, the physician gently asked her for permission to perform a gynecological assessment. She allowed the introduction of Hegar dilators until number 8. She was progressively informed that there were no issues with the inside of her vagina. However, she had a very fibrotic hymen. The couple was offered a simple surgical procedure to facilitate penetration. The procedure resulted in a very adequate and anatomically shaped vaginal introitus. Nevertheless, she was unable to have adequate intercourse, because she still feared feeling pain again. The couple did not accept the idea of starting psychotherapy. IVF was then proposed.
6. MDLN, 37, had been married to a man "in his sixties" for five and a half years and sought help because of vaginismus. They had never had successful intercourse, and he had no children. They came from a very religious background, and the two had been priests. She said she was not sure if she would be able to tolerate transvaginal examination, but she was willing to give it a try. In fact, during IVF treatment she had four TVUS, all performed by two female physicians previously informed of the situation and told to proceed very carefully. During oocyte pick-up her hymen was found to be ruptured. The day after she complained of abdominal pain and headache. However, clinical, abdominal and transvaginal ultrasound examination indicated everything was normal. She had a transfer under sedation of two D3 embryos, but was unable to achieve pregnancy.
7. JSGH, 36, married to a man aged 38, sought help in November of 2011 (when she was 30) because of dyspareunia. An obstetrician-gynecologist associated to our clinic reported she was unable to bear gynecological examination. She was diagnosed with vaginismus and after some time and relaxation therapy, she allowed the introduction of a small speculum, the collection of a sample for a Pap smear, and even TVUS, although only with specific physicians. She managed to have intercourse, but was unable to get pregnant. In November of 2016, the couple had an unsuccessful IUI. Now, they are preparing for IVF.

### B-Questionnaires:

In July of 2016, we sent out questionnaires to 228 centers, of which only 13 replied (5.7%). In January of 2017, the questionnaires were again sent to the centers that had not responded the first time. Eleven replied. A total of 24 answered questionnaires (10.5%) were collected. Another 11 questionnaires never made it to the addressees because of either incorrect/inexistent email addresses or full inboxes.

Fifty percent of the responding centers performed 101-300 IUI and IVF/ICSI cycles a year; 17% did 301-500 cycles; 12% did more than 501 cycles; and 21% fewer than 100 cycles. Most centers stated they offered psychological care to patients undergoing treatment (90%), although further characterization found that 26% offered it routinely, 47% only to select cases, 16% did it randomly, and 11% upon request.

**Figure 1:**
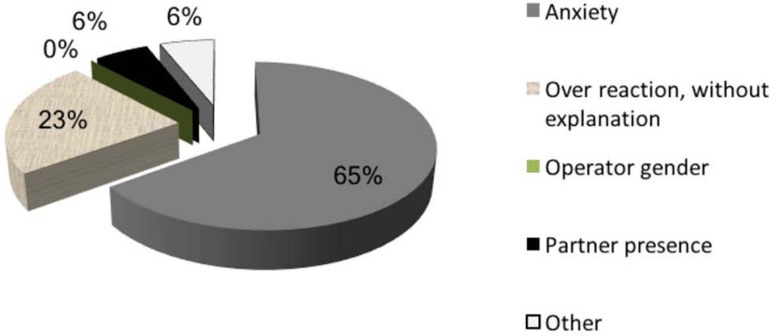
The most likely explanation for trouble with TVUS

Nineteen percent of the centers claimed that none of their patients had trouble undergoing TVUS. Five percent of the centers stated issues occurred with 5-15% of their patients, while 76% of the ART centers reported issues with TVUS examination in less than 4% of their cases. Trouble with TVUS was reported in 61% of the cases by medical staff interviewing patients and in 39% of the cases by ultrasound operators examining patients. Anxiety (65%) was described as the most likely cause of complaints (Figure 1), followed by overreaction (23%). One answer linked complaints to prior procedures for endometriosis.

**Figure 2:**
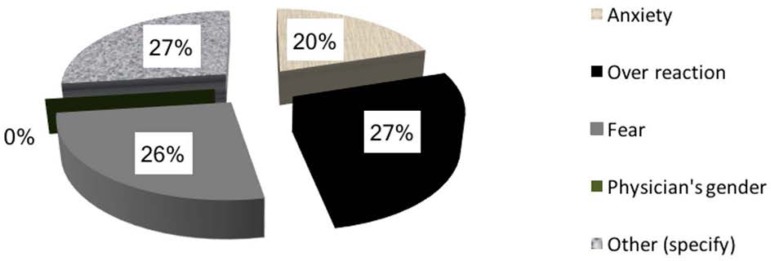
Reasons for requesting sedation in embryo transfer and insemination procedures

Most centers sedated their patients for oocyte pick-up (92%). When asked about sedation during embryo transfer and insemination, 33% said their patients never requested it and 67% characterized it as a rare request. The answers concerning specific situations arising from pick-ups performed on sedated patients were divided into four categories (Figure 2), none of which considering the gender of the physician as a possibility. Four specific answers cited cervical stenosis or trouble inserting the catheter, pain, and extreme anxiety.

The general impression left by the survey was undefined (38%) and intriguing (14%). More than a third (34%) thought the survey was objective and 14% made comments saying they would have liked to know the purpose of it or deemed the survey superficial.

## DISCUSSION

The Lexicon-medicum by Hooper (1817) described dysmenorrhea as difficult or painful menstruation. However, at that time there was no specific reference to vaginismus. It was only in November of 1861 that Dr. J. Marion Sims, an American gynecologist, named and described the syndrome of vaginismus, which linked symptoms of vaginal hypersensitivity to muscle spasms. Dr. Sims concluded that the only rational treatment for the disorder was surgery (Cryle, 2012). Dysmenorrhea and vaginismus are now definitely associated conditions (Araujo & Lotufo Neto, 2013) according to the DCM-5 (Diagnostic and Statistical Manual of Mental Disorders- 5), under the name of genital-pelvic pain/penetration disorder (GPPPD). Today, most of the studies on female sexual dysfunctions report the efficacy of sex therapy based on techniques developed by Masters and Johnson and cognitive-behavioral treatment programs. Although these approaches might be effective for dyspareunia and vaginismus, Staccini (2015) pointed out that more research on GPPPD is needed.

A cross-sectional study was performed in Iran with 236 women referred to the Fatima Zahra Infertility Center of Babol (Bakhtiari *et al*., 2016). The majority of them (84.9%) suffered from primary infertility and had been diagnosed with infertility for a mean of 60.2±8.4 months. The prevalence of sexual dysfunction was 55.5% (n=131). Dyspareunia was found in 28% (n=66), impaired sexual desire and lack of orgasm in 26.3% (n=62), vaginismus in 15.2% (n=36), and lack of sexual stimulation in 13.6% (n=32) of the patients.

Cultural differences aside, our study included seven couples with women holding higher education degrees with a mean age of 37.8 years (husbands with a mean age of 41.8 years). In this cohort they had been married for at least four years, and although two of them said they could tolerate intercourse, all were identified as having had previous treatments for vaginismus, including vaginal dilators and/or speculum (2), psychotherapy (4) or psychiatric assistance, use of anti-depressants or anti-anxiety medication (2).

Interesting facts were captured from the questionnaires sent twice to Brazilian and Latin American ART centers. The low response rate (10.5%) might indicate that trouble performing control ultrasound examination and need to sedate patients for transfer procedures were irrelevant issues in the surveyed centers. However, since 81% of the responding centers agreed that these situations occur, albeit with low incidence, we might be facing an unclear situation. As to the reasons why patients asked for transfers under sedation, anxiety (27%), fear (27%), and overreaction (23%) were cited. However, none of the centers used words such as vaginismus or dyspareunia, or made any reference to sexual dysfunction in a direct way. At one time it was suggested that the cause of the difficulty was prior surgery for endometriosis. Other than that, most centers (90%) claimed they offered psychological care, although multidisciplinary intervention was never clearly defined. It is our belief that we are facing a population whose specific needs are not being properly observed or met. Quality of care and service may be significantly improved once patient demands are considered. After all, according to Dancet *et al*. (2013), patients desire, first and foremost, attention.

The cases presented herein have been more thoroughly analyzed to enhance the perception our teams have had of patient needs. Assuming that our bodies hold the projected feelings of previously experienced psychosomatic suffering, the intense pain stemmed from a disorder might transfigure and resurface in other areas of everyday life. It might not be unlikely for these conscious and unconscious memories to materialize at the time of examination for ART procedures.

The most notable experience occurred when patient n.2, sedated and cleared by the anesthesiologist to start the pick-up procedure, contracted her pelvic muscles to the point of breaking a disposable speculum with a loud bang. This event, witnessed by the psychoanalyst, led to several reflections within the group. Could some of the episodes of vaginismus simply not have an organic cause, for easier clinical labeling? Dolto (2015) considers the possibility that "the body is diseased, but the origin of its functional, physiological disrepair, is an unconscious, psychological disorder."

Tonet (2010) found in women with vaginismus a sometimes extremely rigorous religious upbringing (couple n.6 had been priests and couple 3 expressed important religious concerns regarding the freezing of embryos), in addition to repressive sexual values, feelings of guilt, lack of confidence, fear, and pain (the latter three might be associated with trauma due to misinformation or history of sexual abuse, this last possibility reported by patient n.4).

Some of the traits detected in these patients were suggestive of hysteria. In several texts about the history of hysteria, Freud (1892; 1894; 1896; 1905a;b; 1909) recognized the existence of a psyche in its unconscious determinations, where symptoms had to do with the individual's unique history. According to Freud, hysteria may be seen as a message to be deciphered denoting the existence of conflicts. In recent years, hysteria has been fragmented by psychiatry in several organically and/or neurologically based disorders, by symptom description. There is, nevertheless, a movement favoring the reassessment of clinical data, looking at new and current forms of the disease (Alonso & Fuks, 2004).

In this same line of thought, Nasio (1991) pointed out that the bodily location of hysteria does not exactly obey the law of anatomy or physiology of the body, as clinicians might be inclined to believe. This "body" suffers from its genital parts (with inhibitions like frigidity, impotence, aversion, and ... vaginismus) and its other non-genital parts. In fact these individuals (and women, in our case) are consumed by fear and, to mitigate the ensuing anguish, sustain in life a state of unconscious dissatisfaction. Something related to such unconscious dissatisfaction may be noted when subject n.3 ordered her husband to "look away and shut up."

Women with vaginismus may also have men with more passive or resigned attitudes as their partners, or even men with sexual difficulties (as the husband in case n.4). A more tenuous masculine attitude was observed in case n.1, in which the husband, always quiet and acquiescent, made a point to ask the doctor to have his name written in full and accurately spelled in the examination requests. That was his chance to be been seen and heard, the psychoanalyst reflected.

Therefore, professionals working in ART centers must be aware of the possibility of meeting individuals with GPPPD every time they are faced with "difficult" patients. Couples might hesitate to speak of such matters and cause impediments as treatment progresses. Everyone in the team must be aware of the signs and listen to the details that might foster the discussion of such matters. Questions such as "Is there anything else you think I should know?", "How do you feel about the treatment?", "Would you like to add more information?" and the likes might be used to start new relevant conversations. In four of the seven cases the patients had no trouble discussing the issue with the physician. In two cases it was the nurse who found out about the issue. And in one case the patient described her situation to the psychoanalyst.

Patient n.2, who until then had never had a gynecological exam, would be extremely anxious if she thought a TVUS would be attempted. However this same woman, pregnant and very happy, for the first time in her life had a TVUS. The examination occurred after she discussed with the physician the options to assess the progress of her pregnancy on the seventh week of gestation. She told him to carry on and had a perfect exam. Her husband was with her. At the end of the TVUS, the impression reported to the psychoanalyst was that "she looked complete." Citing Nasio (1991), "there are three conditions when the hysteric patient calms down and gives himself a truce: when he is loving, when he is sad and, when woman, when she is pregnant."

## CONCLUSION

Regardless of the chosen approach, we must be aware of the fact that patients and couples suffering from psychopathological processes and psychic conflict are present in ART centers. The multidisciplinary teams assigned the task of seeing these women and couples must resort to gentle care and sensitive listening so as to provide them with better quality treatment.

## Annex

This is a research questionnaire and your participation is important. Please answer the questions below. Thank you for your participation!

**Table:**

1. How many IUI + IVF/ICSI cycles per year are carried out in your center?	
<100	( )
101-300	( )
301-500	( )
>501	( )
	
2. Is psychological care offered to the patients undergoing treatment at your center?	
Yes	( )
No	( )
	
3. If so, the most appropriate way to characterize the supply of psychological care is:	
Routinely offered	( )
Select cases only	( )
Randomly scheduled	( )
Upon request	( )
	
4. In control ultrasound, how often do patients report trouble with examination?	
Never	( )
Rarely (<4%)	( )
Sometimes (5 to 15%)	( )
Frequently	( )
	
5. Who reported trouble with ultrasound examination?	
Patient	( )
Nurse	( )
Psychologist	( )
Ultrasound operator	( )
	
6. In your opinion, what is the most likely cause of trouble with ultrasound examination?	
Anxiety	( )
Overreaction	( )
Gender of the operator	( )
Presence of the partner	( )
Other (specify)	( )
	
7. Are oocyte pickups for IVF/ICSI routinely performed under sedation at your center?	
Yes	( )
No	( )
	
8. How often do patients ask for sedation in embryo transfer and insemination procedures?	
Never	( )
Rarely (<4%)	( )
Sometimes (5-15%)	( )
Frequently	( )
	
9. To what do you ascribe the requests for sedation in embryo transfer and insemination procedures in your center?	
Anxiety	( )
Overreaction	( )
Fear	( )
Gender of the physician	( )
Other (specify)	( )
	
10. In your opinion, this questionnaire is...	
Irrelevant	( )
Intriguing	( )
Undefined	( )
Direct	( )
Other (specify)	( )
